# Change of Serum Metabolome and Cecal Microflora in Broiler Chickens Supplemented With Grape Seed Extracts

**DOI:** 10.3389/fimmu.2020.610934

**Published:** 2020-12-08

**Authors:** Guangtian Cao, Xinfu Zeng, Jinsong Liu, Feifei Yan, Zhentian Xiang, Yongxia Wang, Fei Tao, Caimei Yang

**Affiliations:** ^1^ College of Standardization, China Jiliang University, Hangzhou, China; ^2^ Key Laboratory of Applied Technology on Green-Eco-Healthy Animal Husbandry of Zhejiang Province, The Zhejiang Provincial Engineering Laboratory for Animal Health and Internet Technology, College of Animal Science and Technology, Zhejiang A & F University, Hangzhou, China; ^3^ Zhejiang Vegamax Biotechnology Co., Ltd., Anji, China

**Keywords:** anti-inflammatory effect, proanthocyanidins, grape seed extract, cecal microflora, serum metabolomics

## Abstract

Grape seed is rich in vitamin E, flavonoids, and proanthocyanidins and has the potential to be used as an antibiotic substitute in broilers. We investigated the effects of grape seed proanthocyanidin extract (GSPE) on growth performance, immune responses, cecal microflora, and serum metabolism in early stage broilers. Data indicated that GSPE improved broiler growth performance by strengthening antioxidant capacity, enhancing immune responses, and increasing cecal short chain fatty acids. 16S rRNA sequencing indicated that GSPE changed the predominant cecal microflora and induced the metabolism of amino acids, lipids, and carbohydrates. An UPLC-Q-TOF/MS-based metabolomics analysis identified 23 serum metabolites (mainly related to lipid, amino acid, and alkaloid) were extremely changed by GSPE treatment. The correlations between the changes of cecal microflora and serum metabolites in birds fed with GSPE were analyzed. Hence, GSPE potentially provides active ingredients that may be used as antibiotic substitute and reduces environmental pollution by grape by-products.

## Introduction

Recently, along the implementation of antibiotic ban in a growing number of countries, plenty of studies have been conducted to seek the antibiotic substitutions in livestock industry. Grape seed is rich in polyphenols (especially proanthocyanidins) and is a natural agricultural by-product with powerful antioxidant and anti-inflammatory effects. Grape seed is an outstanding antioxidative substance, with anti-inflammatory and anti-cancer effects (1, 2). Containing 60–70% of extractable polyphenols, the phenolic compounds from grape seeds comprise flavonoids, as well as catechins and their polymers (3, 4). The valorization of grape seed extract (GSE) could reduce the environmental pollution caused by wine production. It also potentially provides active ingredients for antibiotic substitution. Previous experiments have already revealed the potential applications of GSE in animal nutrition to enhance animal health status and product quality (5, 6).

Grape seed proanthocyanidin extract (GSPE) is enriched with polyphenolic ﬂavonoids, oligomeric proanthocyanidins, and polymerized oligomers (7). Remarkably, previous studies revealed that GSPE has the wide positive effects, including anti-inﬂammatory, cardioprotective, and neuroprotective (8, 9). In addition to antioxidant function, GSPE shows beneficial effects on inflammatory processes (10). Moreover, proanthocyanidins are potential antibacterial compounds with the ability to inhibit the colonization of pathogenic intestinal bacteria (11, 12). Dietary polyphenol-rich grape products were also effective in boosting the development of extraordinary beneficial intestinal microbiota, competitively inhibiting particular harmful bacteria (6, 12).

In fact, increased efforts are now being directed toward a broader revaluation of polyphenol-rich plants for obtaining high-value grape seed by-products. In the mouse model induced by aflatoxin B1 (AFB1), GSPE significantly improved body weight gain, reduced oxidative damage to the spleen, and alleviated immune injury (13). A study by Cardona et al. (14). revealed that a dietary intake of red wine polyphenol increased the intestinal concentrations of healthy gut microbiota (including *Enterococcus*, *Prevotella*, *Bacteroides*, *Bifidobacterium*, and *Blautiacoccoides*). However, it did not change *Lactobacillus* spp. but significantly constrained the development of pathogenic bacteria (such as *Clostridium difficile* and *Clostridium perfringens*) (14). We suspected that there might be a relationship between intestinal microflora and metabolites in broilers supplemented with GSPE. Therefore, the present study was conducted to investigate whether GSPE influences the growth performance, immune response, cecal microflora, and serum metabolites in early stage broilers.

## Materials and Methods

### Test Substances Grape Seed Extracts

Grape (*Vitis vinifera* var. Bobal) seeds were purchased from Champagne (France). The GSE was extracted with water, concentrated, and spray dried. Its components were analyzed using the Agilent 1290 UPLC system (Ultra-Performance Liquid Chromatography) (Agilent, CA, USA) equipped with a binary pump, an auto-sampler, a photodiode array detector, and a column heater. The total polyphenol concentration (expressed as gallic acid equivalents) was determined following the methodology of Chamorro et al. (15). The resultant dry material was 80.5 ± 3.2 g/100 g. The major proanthocyanidins constituents of GSE were analyzed using high-performance liquid chromatography (HPLC). The major constituents were catechin (5.52 ± 0.21 g/100 g), epicatechin (3.14 ± 0.11 g/100 g), procyanidin B1 (1.48 ± 0.06 g/100 g), procyanidin B2 (1.16 ± 0.06 g/100 g), and procyanidin C1 (0.68 ± 0.04 g/100 g). In addition, the GSPE consisted of 4.80% water and 0.435% ash.

### Animal Treatments and Design

A total of 960 AA^+^ one-day-old broilers were randomly divided into four treatment groups (8 replicates, 30 birds per pen). The birds in the control group were fed the basal diet (Control). The birds in the antibiotic group were fed the basal diet with added 20 mg bacitracin/kg (Anti). The birds in the low dose GSPE group were fed the basal diet with 200 mg GSPE/kg (GseL) added. The birds in the high dose GSPE group were fed the basal diet with an addition of 400 mg GSPE/kg (GseH). Throughout the 21-day experiment, all birds were cultivated on floor pens and provided the free access to food and water, with a 23-h photoperiod. In the first week, the room temperature was 35°C and was decreased 2°C each week. The composition and nutrients of basal diet aligned to meet the NRC Nutrient Requirement (2012) (**Table 1**). This study was performed in strict accordance with the Animal Management Rules of the Ministry of Health of the People’s Republic of China and approved by the Animal Care and Use Committee of Zhejiang A&F University (Hangzhou, China).

**Table 1:** Composition and nutrient levels of the basal experimental diet (air dry basis)^1^.

Ingredients	Content (%)
corn	56.33
soybean meal	24.50
fish meal	5.00
extruded-soybean	5.00
limestone	1.30
soybean oil	1.20
corn gluten meal	2.00
fermented soybean meal	1.67
vitamin-mineral premix^2^	3.00
Total	100.00
nutrient levels	% DM
AME (kcal/kg)	2949
crude protein	20.60
crude fat	4.90
lysine	1.17
methionine + cysteine	1.45
threonine	0.87
tyrosine	0.26
calcium	1.00
available P	0.40

^1^AME, apparent metabolizable energy; DM, dry matter.

^2^Supplied per kilogram of diet: vitamin A (retinyl acetate), 1,500 IU; cholecalciferol, 200 IU; vitamin E (DL-α-tocopheryl acetate), 10 IU; riboflavin, 3.5 mg; pantothenic acid, 10 mg; niacin, 30 mg; cobalamin, 10 μg; choline chloride, 1,000 mg; biotin, 0.15 mg; folic acid, 0.5 mg; thiamine, 1.5 mg; pyridoxine, 3.0 mg; Fe, 80 mg; Zn, 40 mg; Mn, 60 mg; I, 0.18 mg; Cu, 8 mg; Se, 0.15 mg.

Representative sample birds were euthanized at day 21. Blood was collected from the jugular vein of the sample birds, 64 birds (4 treatment groups, with 8 replicate pens and 2 birds sampled per pen). Coagulation-promoting tubes were used to obtain the serum; the blood was allowed to stand at room temperature for 4 h and centrifuged at 3,000 g for 15 min. The cecal contents were collected in an aseptic cryopreservation tube and stored below −80° for the detection of SCFAs and microbial sequencing. After gently washing the intestinal content using a sterile PBS, the jejunal and ileal mucosa were wiped on sterile slides, and stored below −80°C for immune factors detecting.

### Growth Performance

All birds per pen were weighted at the beginning and end of every week to calculate the average daily gain (ADG). Moreover, residue feeding and number of deaths were recorded to calculate the average daily feed intake (ADFI) and mortality. The feed:gain ratio (F:G) was calculated using the ADFI and ADG.

### Serum Antioxidant Indexes

The serum antioxidant indexes were tested using commercial kits (Jiancheng Bioengineering Institute, Nanjing, China). The total antioxidant capacity (T-AOC), superoxide dismutase (SOD), glutathione peroxidase (GSH-Px), catalase (CAT), and malondialdehyde (MDA) were determined in accordance with the manufacturer’s instructions.

### Immune Indexes

The serum contents of immunoglobulin A (IgA), immunoglobulin Y (IgY), and immunoglobulin M (IgM), and serum and mucosal interleukin-1β (IL-1β), interleukin-6 (IL-6), and interleukin-8 (IL-8) were detected using specific ELISA kits purchased from Cusabio (Wuhan, China). All methodology followed the manufacturer’s instructions.

### Cecal SCFAs

The concentrations of cecal SCFAs were detected by gas chromatography. Briefly, the SCFA external standards (acetic acid, butyrate, propionic acid, isovalerate, isobutyric acid, and valerate) were obtained from Sigma-Aldrich (Shanghai, China). One gram of cecal content was mixed with 6% phosphorous acid (m/v, 1:3). After vibration and centrifugation, the supernatant was injected into the Agilent Technologies GC7890 Network System (Agilent Technologies, USA), with a column (30 m × 0.25 mm × 0.25 μm, Agilent Technologies) and flame ionization.

### 16S rRNA High Throughput Sequencing

The genomic DNA of the cecal content bacteria was extracted by DNeasy Power Soil Kit (QIAGEN, USA) following the manufacturer’s instructions. The content from both birds sampled in each replicate was mixed into one biological sample. The quality and quantity were determined for the extracted DNA prior to PCR amplification of 16S rRNA genes using the diluted DNA as template. Moreover, due to the bacterial diversity analysis, the V3–V4 variable regions were amplified with normal primers 343F (5’-TACGGRAGGCAGCAG-3’) and 798R (5’-AGGGTATCTAATCCT-3’). The sequencing was conducted at the Illumina Miseq platform from Shanghai OE Biotech Co., Ltd (Shanghai, China)..

Trimmomatic software was used to detect and dissect ambiguous bases, the raw sequencing data were presented in FASTQ format. FLASH software was used to assemble the paired-end reads. Noise reduction of the sequences and removal of reads with chimera were performed utilizing QIIME software (version 1.8.0). The clustering of operational taxonomic units (OTUs) was generated from clean reads using VSEARCH software (version 2.4.2) with a 97% similarity requirement. The entire representative reads selected by the QIIME package were annotated with the SILVA database (Version 123) using a RDP classifier (with a 70% confidence threshold). According to the OTU clustering, a flower plot analysis identified the common and particular OTUs among all treatment samples. The alpha-indexes were used to describe the degree of cecal microfloral diversity within each group; the beta-diversity was analyzed for the difference among all the treatments (using principal component analysis, PCA; Principal Coordinates Analysis, PCoA). Moreover, the linear discriminant analysis (LDA) was coupled with effect size measurements (LEfSe) to distinguish the bacteria between all the treatments, the LDA score was set at 3.5. PICRUSt software (based on the Greengenes data) was applied to predict the composition of the known microbial gene functions. A Kruskal-Wallis test was used to calculate the functional differences between all samples. 

### UPLC-Q-TOF/MS Analysis for Serum Metabolomics

The detection of the serum metabolites in the birds utilized the methodology provided in Li et al. (16). A total of 42 serum samples were analyzed to detect the variation in the bird metabolites, including 12 Control samples and 10 samples of each treatment (GseL, GseH and Anti). An Agilent 6545 Q-TOF/MS system (Agilent Technologies) was used for the chromatographic separation of the serum extract. The UPLC system was equipped with an Agilent Zorbox Eclipse Plus C18 (2.1 × 100 mm, 1.8 μm; Waters Corp., MA, USA). Mobile phases A and B were 95% ACN (containing 10 mM ammonium formate and 1 μl of formic acid) and 50% ACN (containing 10 mM ammonium formate and 1 μl of formic acid), respectively. In the negative ionization mode, mobile phases A and B were 95% ACN (containing 10 mM ammonium acetate, with an adjusted pH to 9 using ammonium hydroxide solution) and 50% ACN (containing 10 mM ammonium acetate, pH 9.0), respectively. The solvent gradient elution in the positive ionization mode involved: 0−2 min, 5% B; 2−20.0 min 5−100% B; 20−25 min, 100% B. The injection volume was 2 μl, and flow rate was set to 0.3 ml/min.

The UPLC system was connected to an Agilent 6545 ESI-Q-TOF high-resolution accurate-mass spectrometer (Agilent Technologies). Both the positive and negative ionization modes were performed. The MS conditions involved: 3.5 kV for positive, capillary voltage; 325°C, gas temperature; 11 L/min, drying gas flow rate; 35 psi, nebulizer pressure; 370°C, sheath gas temperature; 11 L/min, sheath gas flow rate; m/z 100−1,700, mass range. Metabolites were identified using Agilent Mass Hunter Profinder (Agilent Technologies) and the METLIN database (DB).

### Statistical Analysis

SPSS version 24 (IBM Corp.) was used to perform a *t* test and one-way ANOVA analysis to determine significant differences among groups, differences were considered statistically significant at *P* < 0.05, tendency changes but no significant difference were considered at 0.1 < *P* < 0.05. The KEGG data were analyzed using SIMCA-P version 13.0 (Sartorius Stedim Biotech Ltd., Umea, Sweden) for the PCA analysis, and the heat map analysis utilized Multi Experiment Viewer version 4.8 (Boston, MA, USA). The metabolomic data were further analysis by screening the fold change > 2 and *P* value < 0.05 using a *t* test. The metabolism pathway analysis was conducted using MetaboAnalyst 4.0 (http://www.metaboanalyst.ca) and KEGG (http://www.kegg.jp) based on the screened serum metabolites with significant differences (*P* < 0.05). GraphPad Prism 7.0 (GraphPad Software Inc., San Diego, CA, USA) was used to produce the figures.

## Results

### Effects of GSPE on Growth Performance

**Figure 1:**
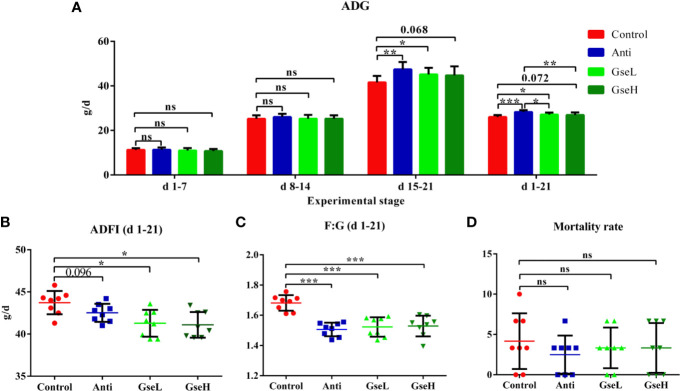
Effects of Anti, GseL, and GseH on **(A)** ADG **(B)**, ADFI **(C)**, F:G, and **(D)** Mortality in broilers from d 1 to 21. *P < 0.05 **P < 0.01, ***P < 0.001, tendency changes but no significant difference were considered at 0.1 < P < 0.05, when compared to the Control treatment. N = 8 per treatment group. ns, not significant.

There were no significant differences in the ADG of birds among all the treatments from d 1 to 7 and d 8 to 14 (**Figure 1A**). Compared with the Control birds, the Anti and GseL birds had significant higher (*P* < 0.05) ADG from d 15 to 21. From d 1 to 21, the ADG of the GseL and Anti birds were higher (*P* < 0.05) than the Control birds. Moreover, the Anti birds had significant higher ADG than the GseL and GseH birds. Furthermore, the supplementation of GSPE significantly decreased (*P* < 0.05) the ADFI of the birds from d 1 to 21 (**Figure 1B**). Both the supplementation of GSPE and antibiotic significantly decreased (*P* < 0.001) the F:G of birds (**Figure 1C**). There was no significant difference between the treatments in bird mortality (**Figure 1D**).

### Effects of GSPE on Serum Antioxidant Indexes

**Figure 2:**
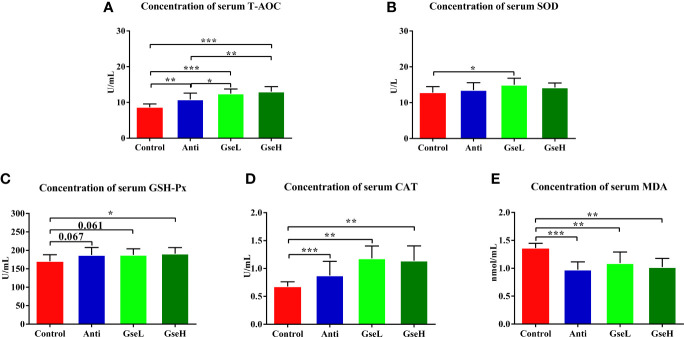
Effects of Anti, GseL, and GseH on the serum contents of **(A)** T-AOC **(B)**, SOD **(C)**, GSH-Px **(D)**, CAT, and **(E)** MDA in broilers on d 21. *P < 0.05 **P < 0.01, ***P < 0.001, tendency changes but no significant difference were considered at 0.1 < P < 0.05, when compared to the Control treatment. N = 16 per treatment group.

The effects of GSPE on the serum antioxidant parameters in early stage broilers are shown in **Figure 2**. The dietary supplementation of the antibiotic and GSPE significantly increased the serum concentration of T-AOC (*P* < 0.01). Supplementation with GSPE significantly (*P* < 0.01) increased the content of serum T-AOC to a higher level when compared to the antibiotic treatment (**Figure 2A**). The level of serum SOD in GseL birds was significant (*P* < 0.05) higher than that in the Control birds (**Figure 2B**). Compared with the Control birds, the GseH-treated birds exhibited significant higher content of serum GSH-Px (**Figure 2C**). Moreover, the supplementation of both the antibiotic and GSPE significantly increased (*P* < 0.01) the content of serum CAT and decreased the MDA of birds (**Figures 2D, E**).

### Effects of GSPE on Serum and Mucosal Immune Indexes

**Figure 3:**
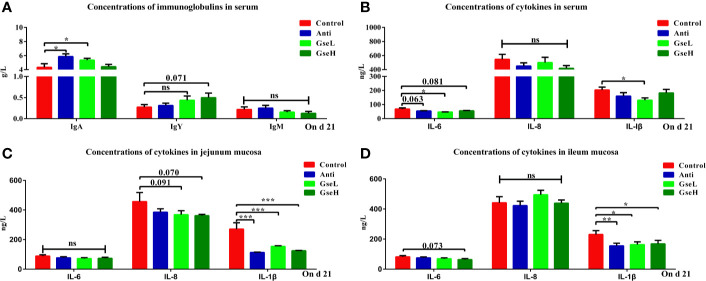
Effects of Anti, GseL, and GseH on **(A)** levels of serum immunoglobulins **(B)**, levels of serum cytokines **(C)**, levels of jejunum mucosal cytokines, and **(D)** levels of ileum mucosal cytokines in broilers on d 21. *P < 0.05 **P < 0.01, ***P < 0.001, tendency changes but no significant difference were considered at 0.1 < P < 0.05, when compared to the Control treatment. N = 16 per treatment group. ns, not significant.

As demonstrated in **Figure 3A**, the levels of serum IgA in the GseL and Anti treated broilers were significantly higher than in the Control birds, while no significant difference was found in the serum IgM regardless of treatments. In addition, the level of serum IgY in the GseH birds was higher (*P* = 0.071) than that in the Control birds, while no significant difference was found within other groups. Compared with the Control birds, the GSPE birds had significantly (*P* < 0.05) fewer IL-6 and IL-1β in their serum (**Figure 3B**). Additionally, the supplementation of Anti (*P* = 0.063) and GseH (*P* = 0.081) decreased the level of IL-6 when compared with the Control group, which there was no treatment effects. Moreover, the contents of ileal and jejunal mucosal IL-1β in birds supplemented with Anti or GSPE were significant lower (*P* < 0.05) than those in the Control birds (**Figures 3C, D**). Compared with the Control birds, although there was no significant difference, the supplementation of GseL (*P* = 0.091) and GseH (*P* = 0.070) decreased the jejunal mucosal IL-6, while only GseH decreased (*P* = 0.073) the ileal mucosal IL-8.

### Effects of GSPE on Cecal SCFAs

**Figure 4:**
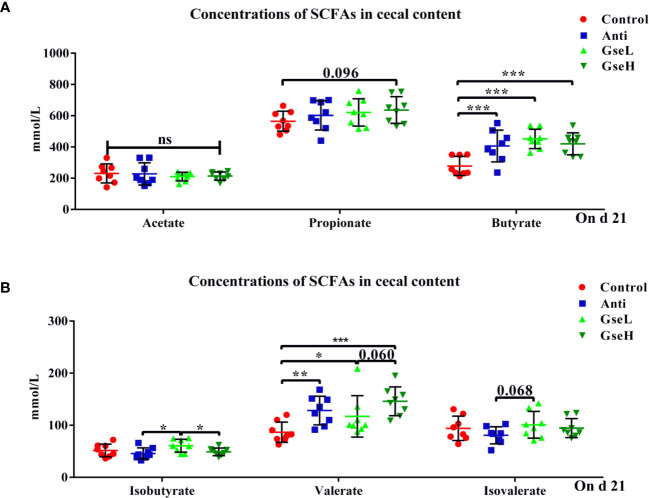
Effects of Anti, GseL, and GseH on the cecal concentrations of **(A)** acetate, propionate, and butyrate **(B)**, isobutyrate, valerate, and isovalerate in broilers on d 21. *P < 0.05 **P < 0.01, ***P < 0.001, tendency changes but no significant difference were considered at 0.1 < P < 0.05, when compared to the Control treatment. N = 8 per treatment group.

The effects of GSPE on the concentrations of cecal SCFAs are shown in **Figure 4**. Both the supplementation of antibiotic and GSPE significantly increased (*P* < 0.05) the concentrations of butyrate and valerate in the colonic content of birds (**Figures 4A, B**). The GseL birds had a significant higher (*P* < 0.05) concentration of isobutyrate than the Anti and GseH birds. In addition, the supplementation of GseH increased (*P* = 0.096) the concentration of propionate to a higher level when compared to the Control treatment, although there was no significant difference. The GseL birds had a higher (*P* = 0.068) concentration of isovalerate when compared to the Anti birds, while there was no significant difference.

### Effects of GSPE on Cecal Microflora

**Figure 5:**
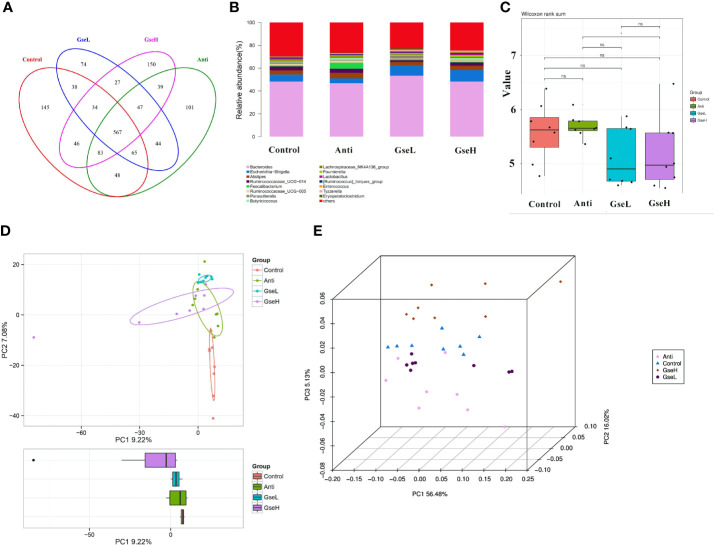
Summary of the microbial community in the cecal contents of broilers on d 21 **(A)**. is a Venn diagram summarizing the numbers of common and unique observed taxonomic units (OTUs) in the microflora **(B)**. is the top 15 relative abundances of bacterial strains (at the genus level) between groups **(C)**. is the Shannon Index reflecting species diversity within and between groups **(D)**. is the PCA plot **(E)**. is the PCoA analysis. N = 8 per treatment group.

Birds in all treatment groups shared 567 OTUs of cecal microflora in the Venn diagram (**Figure 5A**). The Control, Anti, GseL, and GseH birds had 145, 101, 74, and 150 particular OTUs, respectively. Through the relative abundance of top bacterial strains in genus level (**Figure 5B**), *Bacteroides*, *Escherichia-Shigella*, *Alistipes*, *Ruminococcaceae*_UCG-014, *Faecalibacterium*, *Ruminococcaceae*_UCG-005, *Parasutterella*, *Butyricicoccus*, *Lachnospiraceae*_NK4A136_group, and *Lactobacillus* predominated in all birds. There was no significant difference in the alpha-diversity index (only the Shannon index is provided) within all the treatments (**Figure 5C**). Conversely, the beta-diversity analysis using principal component analysis (PCA) showed that the Control samples were the most different to other groups (**Figure 5D**). The PCoA 3D diagram shows samples were clustered within their group, the four treatment groups were well separated from each other (**Figure 5E**).

**Figure 6:**
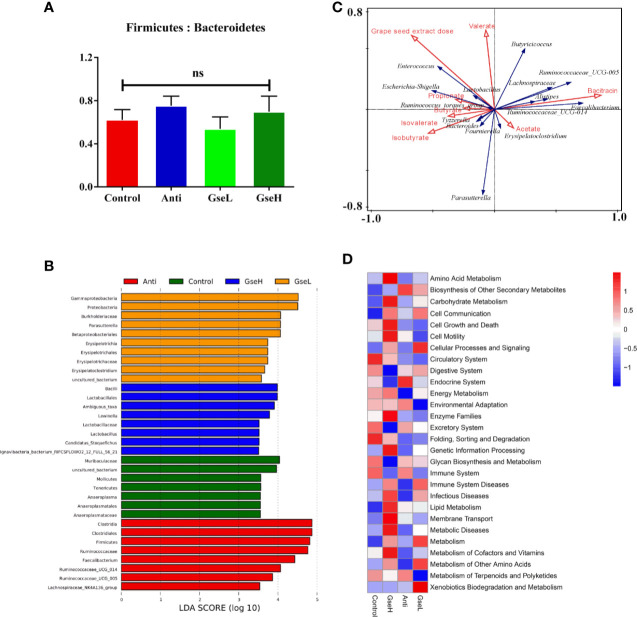
Summary of microbial communities in the cecal contents of broilers on day 21 **(A)**. is the Firmicutes/Bacteroidetes **(B)**. represents the LEfSe analysis **(C)**. is the RDA **(D)**. provides the PICRUSt analysis. N = 8 per treatment group. ns, not significant.

There was no significant difference in the relative abundance ratio of Firmicutes : Bacteroidetes in the cecal microflora within all the groups (**Figure 6A**). The LEfSe analysis indicated that the predominant bacterial strains of GseL were *Gammaproteobacteria*, *Parasutterella*, *Betaproteobacteriales*, and *Erysipelatoclostridium.* The predominant bacterial strains of GseH were *Lactobacillales*, *Lewinalla*, *Lactobacillus*, and *Candidatus-Stoquefichus*. The predominant bacterial strains in the Control group were *Muribaculaceae*, *Mollicutes*, *Tenericutes*, and *Anaeroplasmataceae*. The predominant bacterial strains in the Anti group were *Clostridiales*, *Ruminococcaceae*, *Faecalibacterium*, *Ruminococcaceae*-UCG-005, *Ruminococcaceae*-UCG-014, and *Lachnospiraceae*-NK4A136 (**Figure 6B**). A combined analysis of contents of cecal SCFAs and the top 15 cecal bacteria strains in genus level in the differential dietary treatments, the redundancy analysis (RDA) revealed that the GSPE treatment played a more valuable role in the cecal microfloral variation in birds when compared with antibiotic treatment. The correlations between the GSPE and Anti treatments were negative. Moreover, *Lactobacillus* and the *Ruminococcaceae_torques*_group had the highest correlation with the production of propionate. Meanwhile, the content of isobutyrate and valerate played a positive role in distinguishing the change of cecal microfloral structure (**Figure 6C**). Based on the Greengenes data, PICRUSt analysis showed that the dietary GSPE treatment induced higher heatmap scores in the known functional genes for amino acid metabolism, lipid metabolism, carbohydrate metabolism, cell communication, cellular processes and signaling, energy metabolism, metabolism of cofactors and vitamins, and xenobiotics biodegradation and metabolism when compared to the Control treatment (**Figure 6D**).

### Effects of GSPE on Serum Metabolome

**Figure 7:**
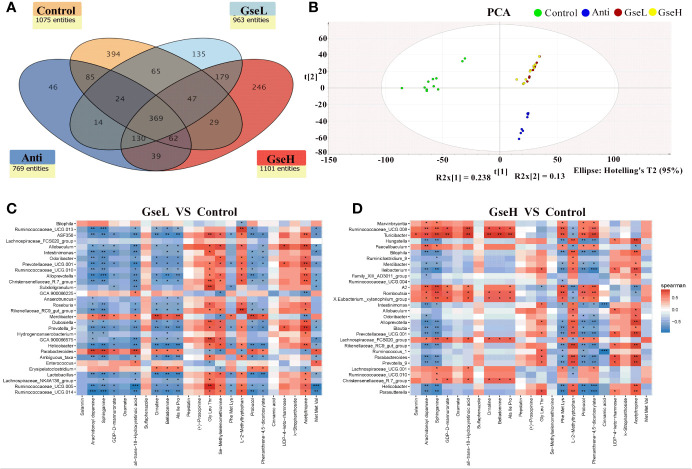
**(A)** is a Venn diagram summarizing the numbers of common and unique observed entities in serum metabolome. **(B)**. represents the PCA plot of the broilers’ serum metabolome. **(C, D)**. represent the correlation between significantly changed serum metabolites and the top 30 distinguished cecal bacteria strains (at the genus level) between the GSPE-treated and Control groups. *P < 0.05 **P < 0.01, ***P < 0.001, compared to the Control treatment. N (Control) = 12; N (Anti, GseL, GseH) = 10.

To investigate the metabolic regulation in GSPE treated birds, the serum metabolites were analyzed using UPLC-Q-TOF/MS. Data showed that 369 identified metabolites were shared among all the treatment groups. There were 394, 64, 135, and 246 metabolites identified in the serum of birds in Control, Anti, GseL, and GseH groups respectively (**Figure 7A**). The Control and Anti samples were well distinguished with those of the GSPE treatment groups using the PCA analysis. There was no definite separation between the GseL and GseH groups (**Figure 7B**). Hence, we hypothesized that the GSE dosage (i.e., 400 mg/kg) was not enough to cause marked changes in the serum metabolites within the GSPE treatment groups.

Moreover, 23 serum metabolites were significantly changed (up-regulated and down-regulated) and detected within all the differential groups after screening with log2 fold change > 2 and *P*-value < 0.05 (**Table 2**). It can be seen that both the dietary GSPE and Anti significantly increased the serum concentration of all-trans-18-hydroxyretinoic acid, arachidonoyl dopamine, sphinganine, and probucol (mainly belonging to prenol lipids and sphingolipid) to higher levels when compared with the Control treatment. The dietary GSPE significantly down-regulated the *k*-strophanthoside related with sterol lipids. The dietary supplementation with both GseL and the antibiotic substantially increased ornaline and oxamate to higher levels when compared with the Control treatment. The GSPE treatment significantly down-regulated the *L*-2-methyltryptophan and pepstatin. The dietary GSE treatment up-regulated the GDP-*D*-mannuronate and UDP-4-*keto*-rhamnose (related with amino sugar and nucleotide sugar metabolism). Moreover, three kinds of alkaloids (acetyltropine, belladonnine, and salannin) were significantly increased by the GseL treatment (*P* < 0.05). One alkaloid [(+)-prosopinine] was significantly decreased by the GSPE and the antibiotic treatment (*P* < 0.05). The GSPE supplementation significantly increased small peptides (Ala Ile Pro and Met Met Val) and decreased Gly Leu Thr and Phe Met Lys. In addition, the dietary supplementation of GSPE and the antibiotic extremely increased the concentration of Se-methylselenomethionine and decreased that of phenanthrene-4,5-dicarboxylate and sulfaphenazole.

**Table 2:** Comparison of the transportation of 23 metabolites across broilers’ serum^1^.

No	Compounds	Formula	Related category	RT (min)	Mass (m/z)	GseL VS Control		GseH VS Control		Anti VS Control	
						Trend	*P* value	Trend	*P* value	Trend	*P* value
1	probucol	C31 H48 O2 S2	lipid modifying agents	7.30	533.3401	up^2^	0.000	up	0.000	up	0.000
2	all-trans-18- hydroxyretinoic acid	C20 H28 O3	prenol lipids	9.95	316.2031	up	0.000	up	0.001	up	0.000
3	arachidonoyl dopamine	C28 H41 N O3	lipids	10.67	439.3139	up	0.000	up	0.000	up	0.000
4	sphinganine	C18 H39 N O2	sphingolipid	12.47	301.2975	up	0.000	up	0.000	up	0.000
5	*k*-strophanthoside	C42 H64 O19	sterol lipids	16.52	872.3828	down	0.000	down^3^	0.000	/	0.873
6	oxamate	C2 H3 N O3	oxamic acid	2.21	106.0363	up	0.003	/^5^	0.930	up^4^	0.032
7	cinnamic acid	C9 H8 O2	phenylalanine	4.56	148.0519	down	0.016	/	0.631	/	0.829
8	*L*-2-Methyltryptophan	C12 H14 N2 O2	amino acid	5.42	218.1071	down	0.000	down	0.000	down	0.022
9	ornaline	C10 H18 N2 O6	nopalinic acid	14.08	279.1419	up	0.000	up	0.047	up	0.020
10	pepstatin	C34 H63 N5 O9	aspartate protease	21.27	702.49	down	0.003	down	0.004	/	0.064
11	GDP-*D*-mannuronate	C16 H23 N5 O17 P2	amino sugar and nucleotide sugar	13.15	636.0884	up	0.000	up	0.010	/	0.896
12	UDP-4-*keto*-rhamnose	C15 H22 N2 O16 P2	amino sugar and nucleotide sugar	14.43	548.0458	down	0.001	down	0.000	/	0.433
13	Ala Ile Pro	C14 H25 N3 O4	peptides	1.23	299.1859	up	0.000	up	0.001	/	0.919
14	Gly Leu Thr	C12 H23 N3 O5	peptides	4.88	306.1934	down	0.002	down	0.001	/	0.775
15	Phe Met Lys	C20 H32 N4 O4 S	peptides	7.66	424.2147	down	0.000	down	0.020	/	0.405
16	Met Met Val	C15 H29 N3 O4	peptides	10.29	401.1434	up	0.000	up	0.000	/	0.898
17	acetyltropine	C10 H17 N O2	tropane alkaloids	13.44	183.1255	up	0.000	/	0.122	up	0.000
18	belladonnine	C34 H42 N2 O4	tropane alkaloids	14.36	542.3187	up	0.000	up	0.001	/	0.898
19	salannin	C34 H44 O9	triterpenoids	16.42	618.2865	up	0.012	/	0.121	/	0.064
20	(+)-prosopinine	C18 H35 N O3	piperidine alkaloids	18.18	313.2615	down	0.000	down	0.000	down	0.000
21	phenanthrene-4,5- dicarboxylate	C16 H10 O4	others	4.38	288.0415	down	0.000	down	0.000	down	0.000
22	Se- methylselenomethionine	C6 H14 N O2 Se	others	11.42	206.0247	up	0.000	up	0.000	up	0.000
23	sulfaphenazole	C15 H14 N4 O2 S	others	14.16	336.068	down	0.002	down	0.003	down	0.009

^1^Control: basal diet; GseL: basal diet + 400 mg GSE/kg; GseH: basal diet + 800 mg GSE/kg; Anti: basal diet + 20 mg antibiotic/kg. ^2^The distinguished metabolite was up-regulated in the GseL group compared to the Control group. ^3^The distinguished metabolite was down-regulated in the GseH group compared to the Control group. ^4^The distinguished metabolite was up-regulated in the Anti group compared to the Control group. ^5^No statistical difference between two treatment groups.

The correlation between the significantly changed serum metabolites and the top 30 distinguished cecal bacteria strains at the genus level is provided in a visual model (as shown in **Figures 7C, D**). We found that sphinganine, all-trans-18-hydroxyretinoic acid, and belladonnine were negatively correlated with the relative abundance of *Ruminococcaceae*_UCG.14, *Ruminococcaceae*_UCG.005, *Lactobacillus*, *Helicobacter*, *Alloprevotella*, *Ruminococcaceae*_UCG.013, and *Intestinimonas.* Conversely, they positively correlated to the relative abundance of *Parabacteroides* and *Merdibacter* in birds that received GseL supplementation. Interestingly, sphinganine, all-trans-18-hydroxyretinoic acid, and belladonnine were positively correlated with the relative abundance of *Ruminococcaceae*_UCG.010, *Christensenellaceae*_R.7_group, *Lachnospiraceae*_UCG.001, *Lachnospiraceae*_FCS020_group, *Romboutsia*, *X.Eubacterium*._xylanophilum_group, and *Ruminococcaceae*_UCG.008 in birds that received the GseH supplementation. Sphinganine was negatively correlated with *Rikenellaceae*_RC9_gut_group, *Prevotellaceae*_UCG.001, *Blautia*, *Alloprevotella*, *Ileibacterium*, *Bilophila*, and *Hungatella* in birds pretreated with GseH.

## Discussion

Currently, our results suggest that dietary supplements of phenolic compounds are likely to improve the health status without affecting productive performance. Proanthocyanidins are natural compounds in plant-based foods (i.e., GSE),. A couple of recently studies also have been conducted to investigate the applications and effects of GSPE in poultry industry (17, 18). To date, adequate evidence exists to support the antioxidation, anti-inﬂammatory, and antimicrobial activity of polyphenols. A previous study conducted by Abu Hafsa and Ibrahim (19) confirmed that grape seed could be treated as an herbal additive agent in the diet of broilers to improve the body weight gain and final body weight (19). GSPE supplementation eliminated the toxic effect of AFB1 on the growth performance of broilers by improving ADFI, ADG, and FCR (13, 19). The present study aligned with previous studies, the addition of GSPE increased the ADG of birds. In addition, the treatment with GSPE decreased plasma ghrelin which normal functions to inhibit food intake and animal weight loss (20, 21). Our results also revealed the supplementation of GSPE markedly decreased the ADFI of birds. Similar to antibiotic treatment, GSPE treatments caused an extreme reduction in F:G. Viveros et al. (12). observed that the supplementation of polyphenol-rich grape pomace extract improved the F:G ratio and nutrition digestibility by increasing the absorptive surface in the intestine of broilers (12). The growth performance of the birds was not influenced by the higher GSPE dose in the present study.

Previous studies revealed that the addition of dietary GSPE could improve the oxidative stress resistance by activating the Nrf2 signaling pathway in broilers (13, 22). Diets containing GSPE (250 and 500 mg/kg) significantly increased the total protein, albumin, and globulin. It decreased the levels of aspartate aminotransferase, alanine aminotransferase, and alkaline phosphatase in the serum of broilers infected with AFB1 (7). We found that the dietary supplementation of GSPE improved the antioxidant levels in birds in the early developmental stage, including increased serum T-AOC, SOD, and GSH-Px and decreased MDA. In addition, the supplementation of commercial grape extracts in broiler chicken diets improved the oxidative stability of the meat (23). It is well known that antioxidative substances derived from plant can greatly reduce damage according to the oxidants by neutralizing free radicals before they attack host cells, and then prevent injuring to proteins, enzymes, and lipids. Dietary grape products might cause an increment in circulating phenolic metabolites by increasing plasmatic α- and γ-tocopherol concentrations, which improves the antioxidant status of broilers (5).

GSPE supplementation significantly ameliorated the serum IL-1β, IL-6, IL-10, and TNF-α. It down-regulated the mRNA expression of serum pro-inflammatory cytokines IL-1β, IL-6, and TNF-α in the spleens of broilers exposed in AFB1 (7). A study by Liu et al. (24). also confirmed that GSE ameliorated host inflammation in mice fed a high-fat diet by markedly decreased the concentrations of serum pro-inflammatory cytokines such as TNF-α, IL-6, and MCP-1 (24). Our results show the supplementation of GSPE to basal diets increased the content of serum IgA and IgY and decreased the content of intestinal IL-1β, and serum IL-6 and IL-1β. GSPE may have changed the mucosal immune system at the jejunum and ileum surfaces by regulating gut microbiota, as suggested by Koren et al. (25). Salinas-Sánchez et al. (26). also speculated that proanthocyanidin derived from GSPE plays an anti-inflammatory role by modulating the microbiota (26). The links between the anti-inflammatory effect and the regulation of intestinal microbiota of GSPE are yet to be elucidated.

The digestive tract contains an abundance of microbial-derived metabolites, which can be easily absorbed through the intestine and play a valuable role in the maintenance of intestinal health (27). Studies confirmed that the SCFAs derived from intestinal microbiota may directly or indirectly affect the unabsorbed nutrients in the intestinal tract (28, 29). As suggested by previous studies, dietary proanthocyanidins can be degraded by intestinal microorganisms into simple phenolic compounds (24, 30). The ingested GSPE was relatively stable in the colon and may affect gastrointestinal tract health (31). GSPE significantly reduced cecal butyrate concentration and modulated the ratio of acetate:propionate:butyrate in mice (32). These studies were in accordance with our findings that the dietary GSPE treatment led to the increment of major colonic SCFAs (butyrate, propionate, and isobutyrate) in present study.

Grape by-products (are extensively debated as antibacterial and feed additives) change the gastrointestinal microflora of broilers (29, 33). Research has confirmed that the flavonoids existing in grape by-products are able to increase the growth of beneficial intestinal bacteria and also restrict certain pathogenic organisms, such as *E. coli*, *Candida albicans*, and *Staphylococcus aureus* (4, 6). Casanova-Martí et al. (32). identified that GSPE changes the diversity of cecal microbiota. This result aligns with the general opinion that polyphenols increase Bacteroidetes and decrease Firmicutes phyla (32). In the present study, the ratio of Firmicutes : Bacteroidetes populations in the GSPE group was not substantially changed; the ratio is normally treated as a marker for intestinal inflammation. Therefore, we undertook a LEfSe analysis and found *Betaproteobacteriales*, *Erysipelatoclostridium*, *Parasutterella*, *Lewinalla*, *Lactobacillus*, and *Candidatus-Stoquefichus* dominated the GSPE treatment. Obviously, the dosage of GSPE influenced the dominant microflora. Other studies produced similar results, dietary polyphenols can help to maintain bowel health by promoting the development of beneficial bacteria (i.e., *Lactobacilli* and *Bifidobacteria*), thus demonstrating the positive prebiotic effect of polyphenols (34). Anhê et al. (35). also confirmed that polyphenols extracted from different plants played a beneficial role in modulating the gut microbiota composition, which increased the abundance of *Bifidobactrium* spp., *Clostridiales*, *Faecalibacterium* spp., *Lactobacillus* spp., and especially the growth of *Akkermansia muciniphila* (35). Some micro-organisms, such as *Lactobacilli*, have been confirmed to use polyphenols compounds in GSE as nutritional substrates to metabolize phenolic compounds for providing energy to cells and effectively affecting bacterial metabolism (12, 36).

The growing evidence reveals that dietary polyphenols from plants including those from grape, tea, and coffee influence the glucose, protein, amino acids, and lipid metabolism *in vitro* and *in vivo* studies (37), which is similar our results to some extent. An earlier study found that GSPE influenced the lipid metabolism by reducing the levels of circulating free fatty acids, limiting the dietary fat absorption and the accumulation of fat in adipose tissue (38). Dietary polyphenols may modulate the glucose metabolism by stimulating peripheral glucose uptake (39). In another study, El-Alfy et al. (40). found that orally administered GSPE decreased the lipid peroxidation and substantially inhibited the rise in blood glucose levels in rats injected with alloxan (40). A rare test was conducted to estimate the change in serum metabolites in GSPE supplemented broilers. Changes in metabolites for lipids, amino acids, alkaloids, and aspartate protease were consistent with previous results in the PICRUSt analysis. Hence, we used MetaboAnalyst 4.0 for analyzing the significantly changed serum metabolites in *Gallus gallus* to identify the related metabolism pathways. Both sphinganine (belonging to the sphingolipid metabolism pathway) and all-trans-18-Hydroxyretinoic acid (belonging to the retinol metabolism pathway) were up-regulated due to the GSPE treatment. Moreover, although no study on the effects of GSPE on serum alkaloids have been reported, the increment of acetyltropine, belladonnine, and salannin and the decrement of (+)-prosopinine were screened for the GSPE treatment. We considered them as the biomarkers for evaluating the influence of dietary GSPE treatment on bird serum metabolites.

GSPE (with their metabolites) reach the colon by enterohepatic recirculation. They were catabolized by colonic microbiota before absorption, generating metabolites in the serum (5). The dietary GSPE encouraged the prevention and treatment of metabolic disorders by targeting intestinal microbiota through a potential prebiotic agent (24). Interestingly, the acetyltropine was oppositely correlated with the bacterial strains related with sphinganine, all-trans-18-hydroxyretinoic acid, and belladonnine. Further study is required to provide a definite explanation. Additional tests should be conducted to investigate the relationship between the digestive microflora and serum metabolome in broilers supplemented with GSPE.

## Conclusion

In summary, we found that GSPE significantly improved the growth performance, increased the serum IgA and IgY concentrations, decreased the mucosal IL-6 and IL-1β, and increased kinds of SCFAs in broilers. Data of 16S rRNA high throughput sequencing of cecal microflora revealed that *Betaproteobacteriales*, *Erysipelatoclostridium*, *Parasutterella*, *Lewinalla*, *Lactobacillus*, and *Candidatus-Stoquefichus* dominated the cecal microflora of birds with GSPE treatment, which higher heatmap scores in the known functional genes for amino acid, lipid, carbohydrate cofactors and vitamins metabolism, cell communication, cellular processes and signaling, and xenobiotics biodegradation and metabolism in GSPE-treated birds from PICRUSt analysis. Based on the non-targeted serum metabolomic method using UPLC-Q-TOF/MS, our statistical analysis found that 13 serum metabolites were significantly up-regulated, and 10 metabolites were significantly down-regulated by the GSPE-supplemented diets in broilers. Additionally, we also identified that all-trans-18-hydroxyretinoic acid and sphinganine may be used as potential biomarkers for evaluating the GSPE regulations. Our findings provide a scientific foundation for GSPE application in broiler feed in the future.

## Data Availability Statement

The original contributions presented in the study are publicly available. This data can be found here: http://www.ncbi.nlm.nih.gov/bioproject/68270; PRJNA682703.

## Ethics Statement

The animal study was reviewed and approved by the Animal Care and Use Committee of Zhejiang A&F University.

## Author Contributions

GC and CY designed the study, conducted the experiments, and wrote the manuscript. FY and YW detected the samples. XZ, JL, ZX, and FT analyzed the results. All authors contributed to the article and approved the submitted version.

## Funding 

We are very grateful for the support of the Natural Science Foundation of Zhejiang Province (No. Q20C170006), the National Natural Science Foundation of China (No. 32002195, 31702153), the Natural Science Foundation of Zhejiang Province (No. LY17C170004), and Basic Technology Research Programs of Zhejiang Province (No. LGN18C200024).

## Conflict of Interest

XZ, JL, and ZX were employed by Zhejiang Vegamax Biotechnology Co., Ltd.

The remaining authors declare that the research was conducted in the absence of any commercial or financial relationships that could be construed as a potential conflict of interest.
